# Rotation Conformational Effects of Selected Cytotoxic Cardiac Glycosides on Their Interactions with Na^+^/K^+^-ATPase

**DOI:** 10.3390/molecules30244815

**Published:** 2025-12-18

**Authors:** Yulin Ren, Peirun Yang, Judith C. Gallucci, Can Wang, Xiaolin Cheng, Sijin Wu, A. Douglas Kinghorn

**Affiliations:** 1 Division of Medicinal Chemistry and Pharmacognosy, College of Pharmacy, The Ohio State University, Columbus, OH 43210, USA; ren.41@osu.edu (Y.R.); gallucci.1@osu.edu (J.C.G.); cheng.1302@osu.edu (X.C.); 2 Wisdom Lake Academy of Pharmacy, Xi’an Jiaotong-Liverpool University, Suzhou 215123, China; ucnvpya@ucl.ac.uk (P.Y.); canwang2004@163.com (C.W.); sijin.wu@xjtlu.edu.cn (S.W.)

**Keywords:** cardenolides, Na^+^/K^+^-ATPase, docking profiles, molecular targets, rotation conformation

## Abstract

Cardenolides are an important group of steroidal natural products and have been used successfully for the treatment of cardiovascular diseases by targeting Na^+^/K^+^-ATPase (NKA) and found more recently to show potential anticancer activity. Biological investigations indicate that both the C-17 lactone unit and the C-3 saccharide moiety of these compounds play an important role in their interaction with NKA and in manifesting the resultant bioactivities. Interestingly, the crystal structures of several cardenolides show various conformations, due to a rotation of the C-3 saccharide moiety or the C-17 lactone unit. These rotation conformations could affect their binding to NKA and the resultant bioactivities, and thus docking profiles with NKA for several cardenolides, including cryptanoside A, digoxin and its aglycone, digoxigenin, and gitoxin, have been investigated in the present investigation. The results indicate that the binding poses of the rotation conformations of the cardenolides selected are different when they bind to NKA, as indicated by their docking scores calculated. For each compound, the rotation conformations observed could be in a dynamic equilibrium, of which each conformer may interact with NKA differentially, and these rotation conformers could act on NKA cooperatively to lead to a specific bioactivity.

## 1. Introduction

Cardenolides are an important group of natural products, of which several representatives have long been used for the successful treatment of congestive heart disease [1]. These products have also attracted wide interest due to their potential anticancer activity, with several compounds having been evaluated in cancer clinical trials, including digoxin and oleandrin [1,2,3,4,5].

Cardenolides bind to Na^+^/K^+^-ATPase (NKA) and inhibit its activity to mediate their own bioactivities [6]. NKA restores Na^+^ and K^+^ electrochemical gradients across the plasma membrane and thus plays a critical role in support of human health [7]. For example, NKA is differentially expressed and regulated by the cardiovascular system and hence has been used as a target for the treatment of cardiovascular disorders [8]. As a signal transducer, NKA binds to Src to form a functional signaling complex [9,10], and thus it also functions as a promising target for the discovery of anticancer agents [11,12]. It is well known that Na^+^ levels are raised in solid tumors and serve as a major contributor to the osmolarity of the tumor microenvironment (TME), which affects immune function [13,14]. Thus, as NKA inhibitors, cardenolides have been reported for their NKA-targeted stimulatory effects on tumor-specific immune responses, indicating their potential contribution to cancer immunotherapy [15,16].

As NKA-specific inhibitors, the binding of cardenolides to NKA depends on the presence of their C-3 saccharide moiety [17]. The crystal structure of a complex of NKA in the phosphoenzyme (E2P) form with digoxin, a representative cardiac glycoside, showed that the 14β and 12β hydroxy groups, the 17β-lactone unit, and the first proximal digitoxose moiety of digoxin all contribute substantially to its interaction with NKA and to its cancer cell line cytotoxicity observed [18,19,20]. Interestingly, the docking scores calculated for digoxin and its derivatives are consistent with their cytotoxic potency [19,20]. Thus, there appears to be a close correlation among the structures, their interaction with NKA, and the bioactivities of digoxin and other cardenolides.

Computer-aided drug design plays an indispensable role in drug discovery, of which molecular docking is used widely for predicting interaction patterns between small molecules and their molecular targets [21]. With this approach, molecular dynamic techniques are used to analyze the physical movements of atoms and molecules and to provide a dynamic and atomistic view of a docking system, and thus molecular dynamics simulations have become useful in the drug development process [22]. However, molecular docking is currently challenged by the treatment of molecular flexibility, and hence incorporating conformational rearrangements of the receptor binding pocket is crucial for effective molecular modeling. Thus far, several methods for improving virtual ligand screening have been developed, including those using multiple fixed receptor conformations [23,24]. In general, the 3D structures of proteins are not stable, and the resultant conformations are involved in various physiological functions. Accordingly, some innovative approaches consisting of mimicking the conformational flexibility of these protein targets have been investigated extensively [25]. As one of a limited number of examples, the impact of the conformational flexibility of aspartame, a dipeptide sweetener, on docking into its receptor has been reported, and the modeling requirements for optimal docking investigations on other small molecules have been discussed. These approaches can support a better understanding of the binding mode of some novel ligands, including ligand and receptor flexibility and the bulk and bridging water molecules [26,27].

**Figure 1:**
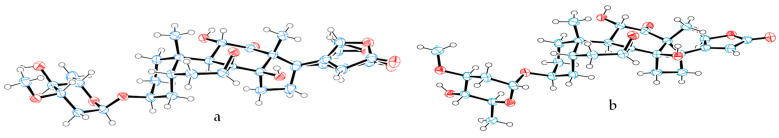
Crystal structures of cryptanoside A showing three different conformations. Two independent molecules (**a** and **b**) were observed in the asymmetric unit. The crystal structure plots were drawn using ORTEP-3 for Windows-version 2020.1. [28], based on its crystal structure reported previously [29]. The ORTEP plots were drawn with 50% probability displacement ellipsoids (oxygen atoms are red, carbon atoms are blue, and the small white circles represent hydrogen atoms, which are drawn with an artificial radius).

**Figure 2:**
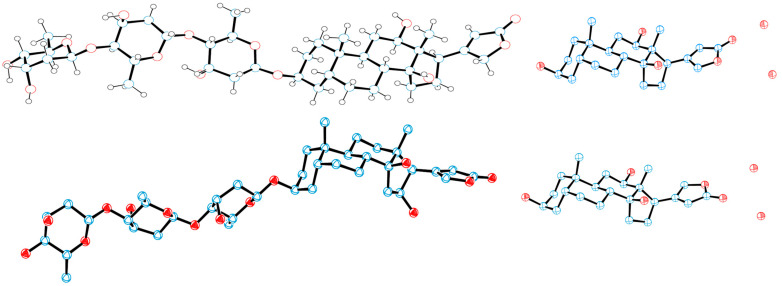
Crystal structures of digoxin (above, left), digoxigenin dihydrate (above and below, right), and gitoxin (below, left) showing different conformations. The crystal structure plots were drawn using ORTEP-3 for Windows-version 2020.1. [28], based on the crystal structure reported previously for digoxin [30], digoxigenin dihydrate [31], and gitoxin [32]. The ORTEP plots were drawn with spheres for all atoms of digoxin and with 50% probability displacement ellipsoids for digoxigenin dihydrate, and gitoxin (oxygen atoms are red, carbon atoms are blue, and the small white circles represent hydrogen atoms, which are drawn with an artificial radius).

The single-crystal X-ray diffraction data collected for the cytotoxic cardiac glycoside epoxide (–)-cryptanoside A (henceforth cryptanoside A) showed that three different conformers exist in two independent molecules (**a** and **b**) in the asymmetric unit. Of these, a disordered C-17 lactone unit was observed in two conformers of molecule **a**, of which the C-3 glycosyl group rotates when compared with that of molecule **b** (Figure 1) [28,29]. These indicate that such a conformational flexibility may occur in other cardenolides. Following this approach, crystal structures of digoxin and its aglycone, digoxigenin, and a close analogue, gitoxin, have been investigated [30,31,32]. The crystal structure of digoxigenin dihydrate showed that the C-17 lactone unit exists in two orientations, with a rotation of 175° [31], while the entire C-3 glycosyl group of gitoxin rotates when compared with that of digoxin (Figure 2) [30,32]. Since a conformational change in aspartame was found to affect binding to its receptor [26], thus various interactions with NKA may occur in the different rotation conformations of a cardenolide. Therefore, the docking profiles for the conformers proposed for digoxin, digoxigenin, gitoxin, and cryptanoside A, based on the rotation of their C-17 lactone unit and C-3 glycosyl group, have been investigated in the present study.

## 2. Results

### 2.1. Overview of the Rotation Conformations of Digoxin and Their Binding to NKA

Digoxin was identified initially from the leaves of *Digitalis lanata* Ehrh., and its different conformations have been elucidated from its NMR spectroscopic and single-crystal X-ray diffraction data [33,34,35]. It is a well-known cardiac glycoside long used for the treatment of congestive heart failure. Interestingly, this compound also shows potential anticancer activity [5], and its cytotoxicity was more potent than that observed for its aglycone, digoxigenin, and a closely similar analogue, gitoxin [36]. Digoxin binds to NKA to mediate its bioactivities, and the binding pose was found to be different from those observed for digoxigenin and gitoxin [20,37], for which all of the C-3 saccharide moiety, the C-12 and C-14 hydroxy groups, the C-17 lactone unit, and the established conformation of the entire molecules could be important [19,20].

**Figure 3:**
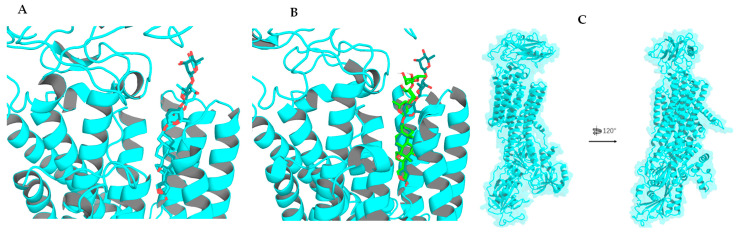
Overlapped docking profiles for digoxin-4RET (deep teal), digoxin-dock, structure of digoxin generated from digoxin-4RET, using a restricted docking box (cyan), or digoxin (**1a**, green) (**A**,**B**) and NKA and the perspective of NKA with an around 120° rotation [(**C**), initial (**left**); rotating left around 120° (**right**)].

**Figure 4:**
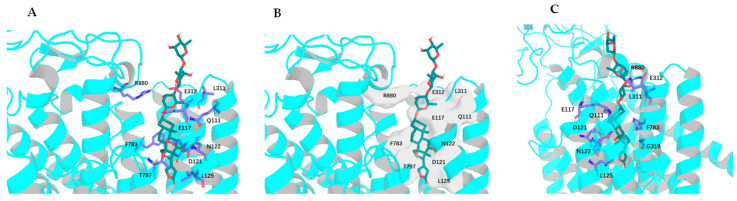
Docking profiles for digoxin-4RET (deep teal) and NKA. The interacting residues are indicated by slate sticks (**A**), gray surfaces (**B**), and slate sticks with rotating the NKA perspective left around 120° (**C**).

To test the effects of rotation conformations of digoxin (**1a**, the conformation shown from the crystal structure of digoxin [30]) on its interaction with NKA, three conformers, **1b**–**1d** [the respective conformations with an around 180° rotation of the C-17 lactone unit (**1b**), the C-3 saccharide moiety (**1c**), and both the C-17 lactone unit and the C-3 saccharide moiety (**1d**) of **1a**], have been docked to the crystal structure of NKA (PDB: 4RET). The modeled structure of 4RET was used as the receptor, and the conformations of **1a**–**1d** and digoxin-4RET, the structure of digoxin shown in the crystal structure of the complex of digoxin and NKA (4RET) [18], generated by LigPrep were used in molecular docking against the receptor by AutoDock Vina [18,20,38,39,40,41]. To present clearly the bending direction and the conformation of the saccharide moiety of digoxin and its analogues and the funnel-shaped architecture of the NKA binding pocket, the perspective of NKA was rotated by 120° (Figure 3). The saccharide moiety of digoxin-4RET exhibits conformational flexibility in the NKA binding pocket, and the 4 Å resolution allows the ligand positioning to be determined in an averaged conformation across multiple states. This indicates that, in a physiological condition, the saccharide moiety of digoxin may be transitioning between a solvent-exposed state and a partially bound form in the inner surface of the NKA binding pocket. In addition, the interacting residues of NKA are presented by stick models and the molecular surfaces to show the specific side-chain conformations and the positions of the residues, respectively, as represented by the docking profiles for digoxin-4RET and NKA (Figure 4).

**Figure 5:**
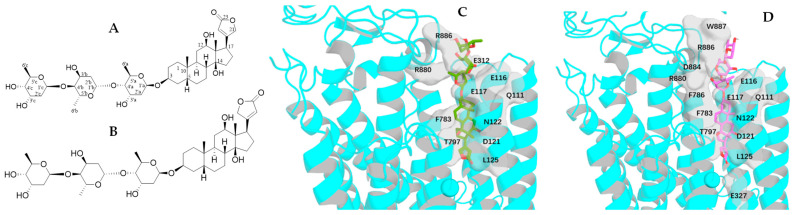
Structures of digoxin (**1a**, (**A**)) and its conformer, **1b** (**B**) proposed, and docking profiles for **1a** (green, (**C**)) or **1b** (magenta, (**D**)) and NKA (the interacting residues are indicated by gray surfaces).

**Figure 6:**
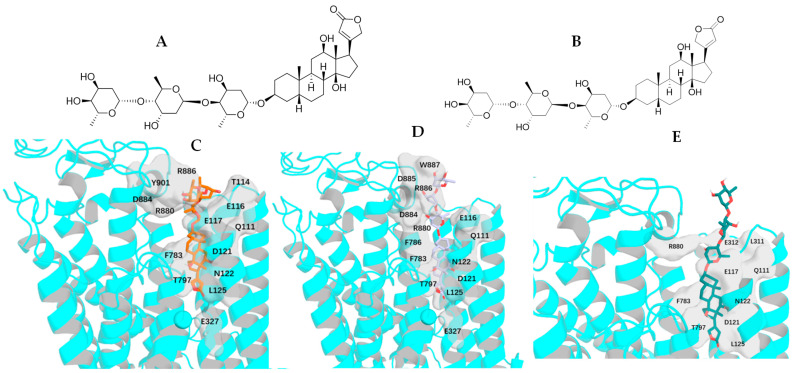
Structures of the conformers **1c** (**A**) and **1d** (**B**) of digoxin proposed, and docking profiles for **1c** (orange, (**C**)), **1d** (gray, (**D**)), or digoxin-4RET (deep teal, (**E**)) and NKA (the interacting residues are indicated by gray surfaces).

The conformers of digoxin (**1a**–**1d**) all contain a steroidal moiety, a C-17β-lactone unit, and a C-3β-glycosyl group. The steroidal core forms a U-shaped *cis*–*trans*–*cis*-fused ring system, which bends inward to build the α-face and protrudes to generate the β-face [30]. Rotation of the lactone unit and the glycosyl group leads to a conformational change, as shown in **1a**–**1d**, which could affect the binding between these conformers and NKA, through changing the docking depth of the aglycone and the binding affinity between the saccharide moiety and NKA in the NKA binding pocket (Figure 5 and Figure 6).

**Figure 7:**
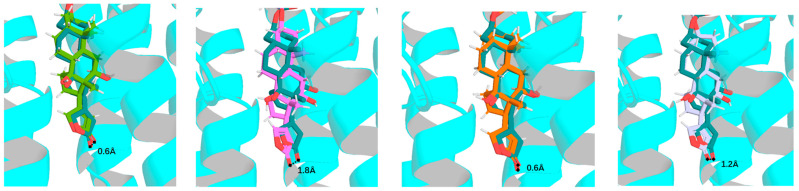
Overlapped partial docking profiles for **1a**–**1d** or digoxin-4RET and NKA showing the aglycone and the distances between O-23 of these conformers and digoxin-4RET (**1a**, green; **1b**, magenta; **1c**, orange; **1d**, gray, and digoxin-4RET, deep teal). The shift between two molecules in the pocket is represented by the distance (the dashed line) of the oxygen atom of the C-23 carbonyl group.

**Figure 8:**
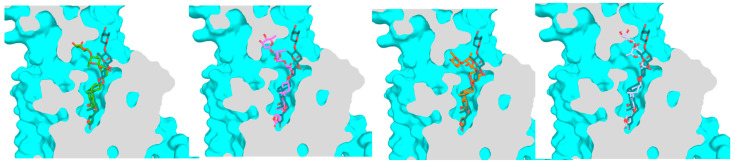
The cross-sectional view of NKA with rotating left around 120° with digoxin-4RET (deep teal) and **1a** (green), **1b** (magenta), **1c** (orange), or **1d** (gray).

The docking profiles showed that the orientation of the steroidal cores of **1a**–**1d** is almost the same, but their docking depth is different, which was indicated by the position of their O-23 (oxygen of the C-23 carbonyl group), with that of digoxin-4RET being used as a reference (Figure 7). Using AutoDock Vina, the docking scores were calculated for the binding of **1a**–**1d** to NKA (PDB entry, 4RET, the complex of NKA E2P-digoxin with bound Mg^2+^ [18]). As shown in the docking profiles, the distances between O-23 of **1a**, **1b**, **1c**, or **1d** and digoxin-4RET were found to be 0.6 Å, 1.8 Å, 0.6 Å, and 1.2 Å, respectively, which was correlated with the docking scores calculated (Figure 7, Table 1). In addition, the second and third glycosyl units of digoxin-4RET are exposed to the solution, and thus the hydrogen bond energy and the increase in entropy within the docking structure are inadequate to offset their solvation energy (Figure 7). Thus, the entire saccharide moieties of **1a**–**1d** could form additional hydrogen bonds or become more dynamic—an increase in entropy to stay bound to NKA. As shown in Figure 8, the aglycones of **1a**–**1d** and digoxin-4RET align, but their saccharide moieties do not. Conformers **1a**–**1d** adopt a conformation that allows them to interact with NKA directly rather than through water-mediated hydrogen bonds, as indicated by the conformation proposed for digoxin-4RET.

### 2.2. Impact of Rotation of the C-3 Saccharide Moiety and C-17 Lactone Unit of Digoxin on Its Binding to NKA

The aglycone of **1a** and digoxin-4RET are almost overlapped in the NKA binding pocket, where an H-bond is formed with the Q111 residue of NKA, with the H-π interaction (hydrogen-π interaction, nonclassical hydrogen bond between a hydrogen atom and an aromatic π system) with the L125 residue of NKA being found to be closely similar for both compounds. However, **1a** seems to shift outward by around 0.3 Å from the pocket, which keeps the lactone unit close to the E117, D121, and N122 residues, to be able to form a stable hydrogen bond. The orientation of the first glycosyl unit of **1a** and digoxin-4RET is partially overlapping, which inclines to form hydrogen bonds with E312 in digoxin-4RET but tends to generate a double-bonded hydrogen bond with the R880 residue in **1a**. The second glycosyl unit of digoxin-4RET forms hydrogen bonds with the L311 residue, but its third one is in solution, with no interaction with NKA observed. Differentially, these saccharide units of **1a** fold inward to form hydrogen bonds with the E116 and R886 residues, with no interactions with the L311 residue (Figure 5).

The major interactions between digoxin-4RET or **1b** and NKA are almost the same, for which H-π interactions with the F783 residue and hydrogen bondings with Q111, D121, N122, E117, G319, and R880 were observed. The C-14 hydroxy and C-18 methyl groups of digoxin-4RET form hydrogen bonds with the D121 and N122 side chains, but these same H-bonds observed for **1b** are relatively weaker when reaching deeper into the pocket to keep the C-14 hydroxy group in the same polar cavity. Compared with digoxin-4RET, the C-17 lactone unit of **1b** extends deeper into the NKA binding pocket to support H-π interactions and H-bonding with the L125 and E327 residues, respectively. Its C-3′a hydroxy group forms hydrogen bonds with the R880 and D884 residues, and its C-5′a methyl group forms another H-bond with the residue E116, while its C-3′b hydroxy group orients towards the residue E116 to form a bidentate hydrogen bond (Figure 5). In addition, a polar surface is formed by the amino groups on the backbone and the side chains of the R886 and W887 residues of NKA, where an H-bonding network is generated by the third glycoside unit of **1b**, which led to a better docking score, when compared with **1a** (Table 1).

The binding site of NKA is a narrow funnel-shaped hydrophobic pocket, with a broad entrance being formed by the T114, R886, Y901 residues and with a narrow bottom being located at the residue E327. In this pocket, binding to NKA with the C-3 glycoside moiety of **1c** and digoxin-4RET is different, but it is almost identical for their aglycone to dock to the pocket (Figure 6). The C-3′a hydroxy group of **1c** shares the same cavity with digoxin-4RET and forms a hydrogen bond with the E327 residue, while its C-6′a methyl group shifts towards the residue R880 to form a bidentate hydrogen bond. The second glycosyl unit of **1c** bends to the opposite direction of the E312 and R880 residues to generate multiple pairs of hydrogen bonds with the T114, E116, and E117 residues, while its third glycosyl unit binds to the positions closer to the inner wall of the pocket and binds to NKA deeply. These support **1c** as having more interactions with NKA, including multiple hydrogen bonds formed with the residues D884, R886, and Y901 of NKA. In addition, the C-3 glycosyl moiety of digoxin-4RET and **1b** bind vertically to one side of the funnel in the NKA binding pocket, but the saccharide unit of **1c** binds to NKA horizontally in the inner surface of the funnel (Figure 5 and Figure 6), which also supports more entropy increase and hydrogen bonds to stabilize its glycosyl moiety.

Compared with digoxin-4RET, **1d** has more interactions with NKA. It reaches deeper into the NKA binding pocket, with its O-23 being closer to the E327 residue, while its C-14 hydroxy group shifts around 1.2 Å into the cavity to weaken hydrogen bonds with the D121 and N122 residues. The C-3′a hydroxy group of **1d** forms multiple hydrogen bonds with the R880 and D884 residues, and its C-5′b methyl group forms a hydrogen bond with the residue V881, while its C-5′a methyl group is distant from Q111, E116. In addition, the second glycosyl unit of **1d** directs closely to the cavity consisting of the R880 and D885 residues, and its third one extends outwards to enable the C-3′c and C-4′c hydroxy groups to interact with the D885 and W887 residues through H-bonds (Figure 6).

### 2.3. Impact of Rotation of C-17 Lactone Unit of Digoxigenin on Its Binding to NKA

**Figure 9:**
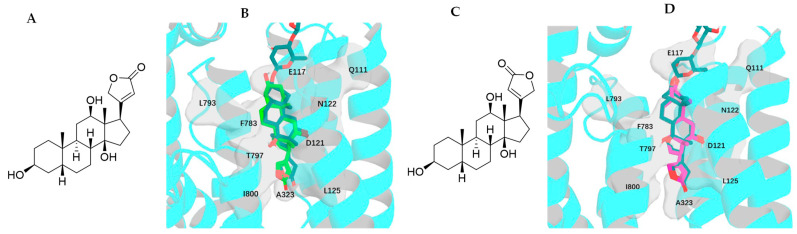
Structures of conformers of digoxigenin, **1e** (**A**) and **1f** (**C**), and overlapped docking profiles for **1e** (green), **1f** (magenta), or digoxin-4RET (deep teal) and NKA (**B**,**D**).

Two conformers of digoxigenin, **1e** (the conformation shown from the crystal structure of digoxigenin [31]) and **1f**, the conformations with an around 180–rotation of the C-17 lactone unit of 1e, are yielded, and their major interactions with NKA are found to be consistent with those observed for the aglycone of digoxin-4RET. These include the H-bonds and the H-π interactions between **1e**, **1f**, or digoxin-4RET and the Q111, E117, D121, N122, G319, and T797 residues and the F783 and T797 residues of NKA, respectively (Figure 9). However, multiple hydrogen bonds are formed between the C-3 glycosyl moiety of digoxin-4RET and NKA, which could contribute to the lower docking score of −11.874 kcal/mol calculated for digoxin (**1a**), when compared with those of −10.875 and −10.446 kcal/mol for **1e** and **1f**, respectively (Figure 9, Table 1). In addition, the C-17 lactone unit of **1e** links to the I800 residue to form an additional H-bond, and this may contribute to its somewhat lower docking score when compared with that calculated for **1f** (Table 1).

### 2.4. Impact of Rotation of the C-3 Saccharide Moiety and the C-17 Lactone Unit of Gitoxin on Its Binding to NKA

**Figure 10:**
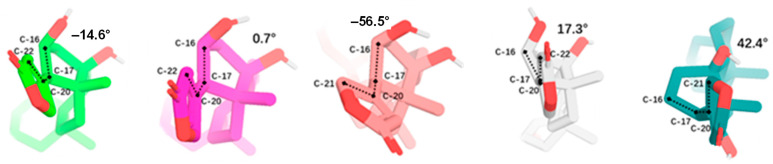
Dihedral angles calculated from the aglycone of **2a**–**2d** or digoxin-4RET. These angles were calculated from C-16, C-17, C-20, and C-21 or C-22, of which C-21 or C-22 was selected, in being closer to C-16. The angles toward the α-face of the steroidal core were defined as being positive, with those toward the β-face of the steroidal core defined as being negative (**2a**, green; **2b**, magenta; **2c**, salmon; **2d**, gray; and digoxin-4RET, deep teal). The dihedral angle and four atoms involved are represented by the dashed lines and black dots.

Four conformers of gitoxin (**2a**–**2d**) are generated either from the crystal structure or from an around 180° rotation of its C-3 saccharide moiety and/or the C-17 lactone unit. Of these, the conformer **2a** is obtained from the crystal structure of gitoxin [32], and **2b** and **2c** are the respective conformations with an around 180° rotation of the C-17 lactone unit and the C-3 saccharide moiety of **2a**. In turn, **2d** is the conformation with an around 180° rotation of both the C-17 lactone unit and the C-3 saccharide moiety of **2a**. During the docking calculation, the computer program tends to draw the structures that could be stable and have more chances of interacting with NKA. Thus, the docking profiles for **2a** and **2b** were found to be almost overlapped, with their respective docking scores of −10.485 and −10.403 kcal/mol being closely comparable. Also, the docking scores calculated for **2c** (−11.245 kcal/mol) and **2d** (−11.261 kcal/mol) are similar (Table 1). These indicate that rotation of the C-17 lactone unit of gitoxin may be unstable, due to the steric hindrance from the C-16 hydroxy group. This has been supported by the proximity between C-21, C-22, and C-16, for which none of **2a**–**2d** can adopt an angle similar to that of digoxin-4RET in the NKA docking pocket (Figure 10).

**Figure 11:**
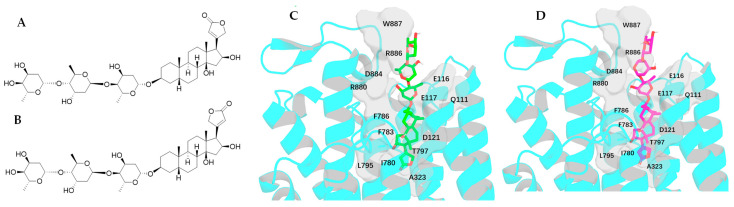
Structures of gitoxin (**2a**, (**A**)) and its conformer, **2b** (**B**), and docking profiles for **2a** (green, (**C**)) or **2b** (magenta, (**D**)) and NKA (the interacting residues are indicated by gray surfaces).

The major interacting residues for **2a**, **2b**, and digoxin-4RET are almost the same, including the hydrogen bonds with the Q111, E117, D121, and T797 residues and the H-π interactions with the residue F783 formed by their steroidal core and the H-bond between their 3′a hydroxy group and the R880 residue. In addition, the C-17 lactone units of **2a** and **2b** shift towards the residue of I780 to form some new interactions with the residues of I780, A323, L795, E116, R886, and W887 (Figure 11).

**Figure 12:**
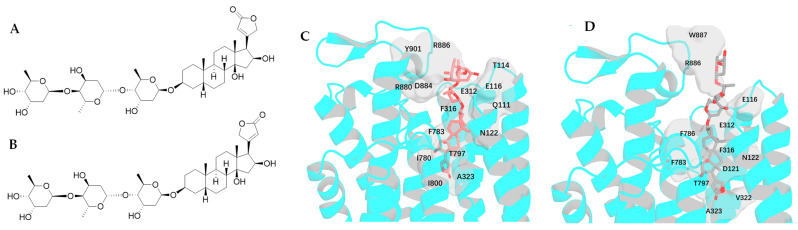
Structures of the conformers **2c** (**A**) and **2d** (**B**) of gitoxin and docking profiles for **2c** (salmon, (**C**)) or **2d** (gray, (**D**)) and NKA (the interacting residues are indicated by gray surfaces).

The aglycone of **2c** shifts downward overall and thus can reach a deep position in the NKA binding pocket, which forms hydrogen bonds with the I780 and A323 and the N122 and T797 residues and H-π interactions with the I800, N122, and T797 residues. Its 3′a hydroxy group can share the same cavity as that for digoxin-4RET to form an additional hydrogen bond with the E312 residue, while its C-6′a methyl group shifts towards the residue R880 and forms a bidentate hydrogen bond (Figure 12). Similar to **1c**, the third glycosyl moiety of **2c** reaches the left side of the NKA binding pocket to form multiple hydrogen bonds with the D884, R886, and Y901 residues (Figure 6 and Figure 12 and Figure S4, Supporting Information). These could all contribute to its better docking score of −11.245 kcal/mol as observed when compared with **2a** or **2b** (Table 1). Compared with **2a** or **2b**, **2d** can dock to the NKA binding pocket deeply to form more hydrophobic interactions with NKA, as well as the hydrogen bonds with the D121, N122, and T797 residues. Also, H-bonds with the residue V322 and H-π interactions with the residues of A323, F316, and F783 were found for their aglycone and additional H-bonds between their first saccharide unit and the E312 residue and between their second glycosyl unit and the E116 residue are generated, with an H-bond network with the R886 and W887 residues being established by their third glycosyl unit (Figure 11 and Figure 12). These interactions all contributed to the docking score of −11.261 kcal/mol calculated for **2d**, which was improved when compared with those for **2a**, **2b** (Table 1).

### 2.5. Impact of Rotation of the C-3 Saccharide Moiety and the C-17 Lactone Unit of Cryptanoside A on Its Binding to NKA

Five conformers of cryptanoside A (**3a**–**3e**) may be generated either from their crystal structures or from rotation of the C-17 lactone unit or the C-3 saccharide moiety. Structures of **3a** and **3b** were drawn based on their crystal structures [29], of which **3b** represents the conformation with an around 20° rotation of the C-17 lactone unit of **3a**. While **3c** and **3d** are the respective conformations with an about 180° rotation of the C-3 saccharide moiety and the C-17 lactone unit of **3a**, and **3e** is the conformation with an around 180° rotation of both the C-17 lactone unit and the C-3 saccharide moiety of **3a**. As shown in Table 1, the docking scores of **3a**–**3c** were found to be much larger than that of **3d**, **3e**, and other conformers proposed for digoxin, digoxigenin, and gitoxin, due to their different binding poses.

**Figure 13:**
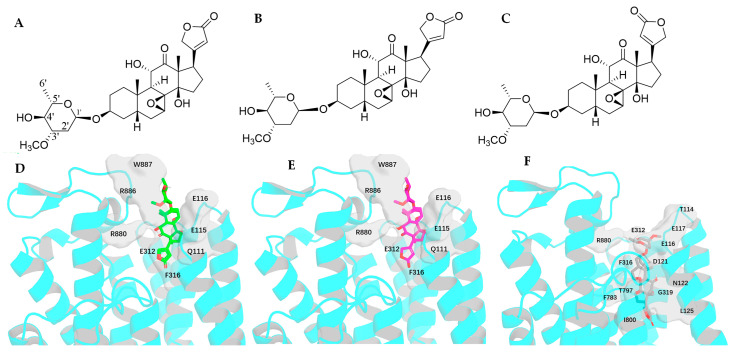
Structures of cryptanoside A (**3a**, (**A**) and its conformers, **3b** (**B**) and **3d** (**C**), and docking profiles for **3a** (green, (**D**)), **3b** (magenta, (**E**)), or **3d** (gray, (**F**)) and NKA (the interacting residues are indicated by gray surfaces).

**Figure 14:**
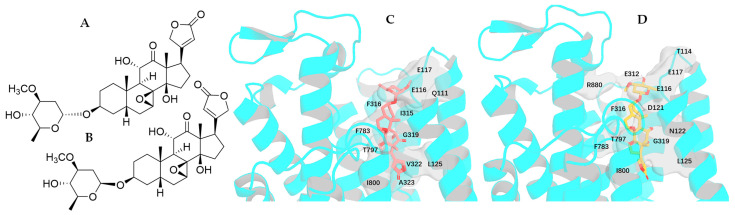
Structures of the conformers **3c** (**A**) and **3e** (**B**) of cryptanoside A and docking profiles for **3c** (salmon, (**C**)), or **3e** (yellow, (**D**)) and NKA (the interacting residues are indicated by gray surfaces).

With around a 20° rotation of the C-17 lactone unit, **3b** is closely similar to **3a**, in which the C-23 carbonyl group is proximate to the C-14 hydroxy group. These conformers can form hydrogen bonds with the Q111, E115, E116, E321, and R880 residues and the H-π interactions with the F316 residue. However, when compared with digoxin-4RET, the C-7 and C-8 epoxide and the C-12 carbonyl group reduce the flexibility of the steroidal core of cryptanoside A, which prohibits **3a** and **3b** from reaching the narrow binding pocket of NKA deeply. Thus, the binding positions of **3a** and **3b** seem to be too shallow to enable them to form more interactions with NKA, with only limited binding to the upper part of the NKA binding pocket being found, including the H-bonds between their 3′a- and 4′a-hydroxy groups and the respective R886 and W887 residues (Figure 13). In the NKA binding pocket, the positions of **3c**–**3e** resemble that of digoxin-4RET and are much deeper than those observed for **3a** and **3b**. The docking profiles for **3c**, **3d**, or **3e** and NKA are partially overlapped, of which, however, a few minor differences could contribute to their varied docking scores (Table 1). The steroidal core and the C-17 lactone unit of **3d** form H-π interactions with the residues of F316 and F783 and the residues of I800, L125, and A323, respectively, while hydrogen bonds are formed between its C-11 and C-14 hydroxy groups and the respective G319 and T797 residues. In addition, multiple hydrogen bonds have been established between its glycosyl moiety and the Q111, E116, and I315 residues (Figure 13 and Figure 14).

The C-17 lactone unit of **3c**–**3e** orients in each case to the C-12 carbonyl group, which is proximate to the α-face in **3c** but to the ß-face in **3d** and **3e**. Similar to **3c**, both **3d** and **3e** form H-bonds with the residue of E116 and H-π interactions with the L125, I800, F316, and F783 residues. However, at variance from **3c**, more interactions with NKA are evident for **3d** and **3e**, due to the different orientations of their C-11 hydroxy and C-12 carbonyl groups. Hence, the C-12 carbonyl group of **3d** forms a hydrogen bond with the G319 residue, and its C-11 hydroxy group generates another hydrogen bond with the residue of T797. Also, both **3d** and **3e** can form additional hydrogen bonds with the T114, E117, D121, N122, E312, and R880 residues (Figure 13 and Figure 14). Thus, **3d** and **3e** each show a better docking score, when compared with **3a**–**3c** (Table 1).

**Figure 15:**
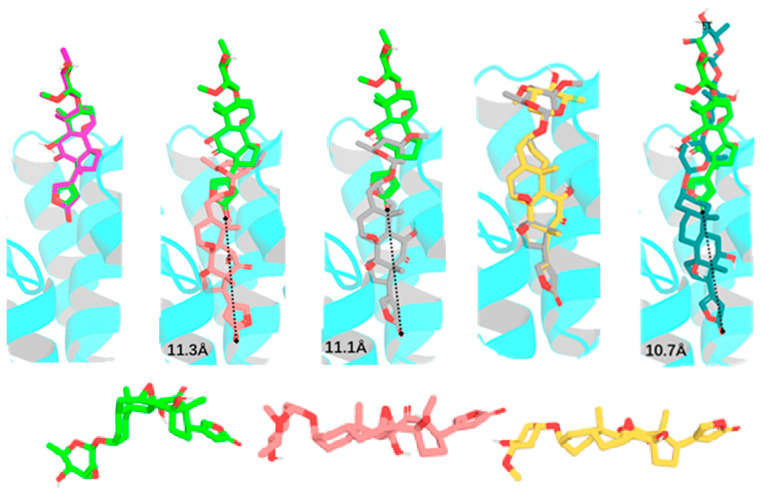
Overlapped docking profiles for **3a** (green), **3b** (magenta), **3c** (salmon), **3d** (gray), **3e** (yellow), or digoxin-4RET (deep teal) and NKA and conformations of **3a** (green), **3c** (salmon), and **3e** (yellow) obtained from their docking to NKA. The shift in depth between two molecules in the pocket is represented by the distance (the dashed line) of the oxygen atom of the C-23 carbonyl group.

The NKA active binding pocket is narrow and elongated, and all of the steroidal core, the lactone unit, and the saccharide moiety of a cardenolide need to align to bind to it efficiently. However, the C-1′–C-3 bond in **3a** and **3b** pivots toward the upside of the glycosyl ring to keep it oriented away from the steroidal core. As a result, the entire molecules of **3a** and **3b** are not aligned, which, in turn, could reduce their binding to NKA. Differentially, the C-1′–C-3 bond in **3c**–**3e** points downward from the glycosyl unit to allow it to refold back onto the axis of the steroidal core and the lactone unit (Figure 15). Thus, these conformers (**3c**–**3e**) can dock to the NKA binding pocket deeply, with reduced steric hindrance.

## 3. Discussion

Cardenolides have been reported to target NKA to mediate their various bioactivities, to which both the C-3 saccharide moiety and the C-17 lactone unit contribute substantially [42]. Interestingly, the cytotoxic potency of these compounds seems to be consistent with their docking scores calculated from their binding to NKA [42,43], indicating that any changes at the C-3 and C-17 positions may affect their binding to NKA and the resultant biological effects.

Rotation of the C-3 saccharide moiety and/or the C-17 lactone unit of several cardenolides has been supported by their crystal structures. For example, three conformers of cryptanoside A were observed, due to rotation of the C-17 lactone unit and the C-3 glycosyl group [29] (Figure 1). An around 175° rotation of the C-17 lactone occurred in the crystal structures of digoxigenin dihydrate [31]. However, no rotation conformers were displayed in the crystal structures of digoxin and gitoxin [30,32], while the entire C-3 glycosyl group of gitoxin rotates when compared with digoxin (Figure 2) [30,32]. Rotation of the C-17 lactone unit of gitoxin may be hindered by its C-16 hydroxy group, as supported by the proximity calculated between C-21, C-22, and C-16 of **2a**–**2d** when they were docked to the NKA active binding pocket (Figure 10). This steric hindrance may also affect the conformation of its C-3 glycosyl group, which is different from that of digoxin. In addition, an around 180° rotation of the C-17 lactone unit of cryptanoside A was not evident in its crystal structures, which may be hindered by its C-12 carbonyl group [29] (Figure 1). Thus, the C-12 and C-16 hydroxy groups of cardenolides could affect the rotation of the C-17 lactone unit, and such an impact may result from both steric hindrance and the potential formation of hydrogen bonding and H-π interactions between these hydroxy groups and the enone moiety of the lactone unit. As a result, the conformations of a cardenolide could be determined based on the crystal structure reported for itself or for its closely similar analogues.

The narrow and funnel-shaped binding pocket of NKA, as shown in the crystal structure of the complex of digoxin and NKA (4RET) [18], indicates that the rotation conformations of cardenolides may affect their binding to NKA. Thus, the binding poses and the resultant docking scores of the rotation conformers present can be different. When compared with digoxin (**1a**), an around 180° rotation of the C-17 lactone unit enables **1b** to dock to the NKA binding pocket deeply to support all of its interactions with NKA, with an H-bonding network being generated between its third glycosyl unit and the polar surface on the backbone of NKA. As a result, **1b** showed a better docking score than **1a** (Table 1). Similarly, an around 180° rotation of the C-3 saccharide moiety supports **1c** in being able to dock to the NKA binding pocket more efficiently. This saccharide moiety of **1c** forms an elaborate hydrogen-bonding matrix with residues T114, E116, E117, D884, R886, and Y901, which represents a substantial enhancement over the limited solvent-exposed interactions as observed in the digoxin-4RET. In addition, different from digoxin-4RET and **1b**, of which the C-3 glycosyl moiety binds vertically to NKA, the C-3 glycosyl unit of **1c** binds to NKA horizontally. This binding pose supports **1c** as having a greater entropy increase and the resultant amount of hydrogen bonding when it binds to NKA (Figure 5 and Figure 6). Thus, the conformers **1b**–**1d** all showed a better docking score than **1a** (Table 1). However, the difference in docking scores found for **1c** and **1d** was much less than that observed for **1a** and **1b**, indicating that the C-3 glycosyl moiety could affect the effects of rotation of the C-17 lactone unit on the binding of digoxin to NKA. This can be supported by the docking scores calculated for its aglycone and digoxigenin (**1e** and **1f**), which are closely similar but much larger than those observed for **1a**–**1d** (Table 1), due to the absence of the C-3 saccharide moiety.

Docking scores calculated for **2a** and **2b** were greater than those observed for **2c** and **2d**, which were found to be closely similar to those for **1b**–**1d** (Table 1). This indicates that an around 180° rotation of the C-3 glycosyl moiety could improve the interaction between gitoxin and NKA, even though such a rotation is not evidenced in its crystal structure (Figure 2). However, the docking scores for **3a**–**3c** were much larger than those observed for **3d** and **3e**, which were similar to those for **1b**–**1d** (Table 1). This suggests that an around 180° rotation of the C-17 lactone unit could be important for cryptanoside A to bind to NKA, even though such a rotation may be hindered by the C-12 carbonyl group, as indicated by its absence in the crystal structures of cryptanoside A (Figure 1).

In the NKA binding pocket, the positions of **3a** and **3b** are shallow, due to their C-7 and C-8 epoxide and the C-12 carbonyl group, which reduces the flexibility of the steroidal core. Also, orientation of their C-1′–C-3 bond keeps the glycosyl moiety oriented away from the steroidal core to decrease the molecular alignment. Hence, both **3a** and **3b** can only bind to the upper part of the NKA. However, the positions of **3c**–**3e** in the NKA binding pocket are much deeper than those of **3a** and **3b**, which allow them to have more interactions with NKA (Figure 13, Figure 14 and Figure 15). These indicate that modifications through changing the conformation of the C-17 lactone unit of cryptanoside A may improve its binding to NKA and the resultant NKA-targeted bioactivities.

Interestingly, the lipid chromatographic and spectroscopic data showed that cryptanoside A is chromatographically and spectroscopically pure, even though its crystal structures showed three different conformers [29]. This suggests that the rotation conformations of cryptanoside A could exist in an equilibrium status in solution, where its C-3 glycosyl group and C-17 lactone unit rotate continuously. Hence, its interaction with NKA and the associated bioactivities could result from a cooperative action of all of the rotation conformers occurring. In addition, the cross-sectional view of NKA rotating left around 120° with digoxin-4RET and the conformers of digoxin, **1a**, **1b**, **1c**, or **1d**, showed that the C-3 saccharide moeity of digoxin-4RET orients differentially in the NKA binding pocket, when compared with **1a**–**1d** (Figure 7). These overall observations indicate that the conformation of a cardenolide may vary when it docks into the NKA binding pocket.

Conformation plays a dominant role in the binding mechanisms [44], and rotational conformations could function as a molecular modulator for exploring diverse binding modes. Rotation of the C-17 lactone unit or the C-3 glycosyl moiety of digoxin and its analogues can generate distinct conformations to modulate their binding to NKA. Thus, the rotation conformations should be considered in molecular modeling, not only for cardenolides and NKA but also for other small molecules and their macromolecular targets.

## 4. Materials and Methods

### 4.1. Compounds and Biological Evaluation

Cryptanoside A, digoxin, and digoxigenin were purified and characterized structurally in previous work by our group, and their cancer cell cytotoxicity and activity at certain molecular targets have been reported [5,9,10,11]. Gitoxin was selected from the literature, with its bioactivities having been published [17,36].

### 4.2. ORTEP Plotting

Based on the crystal structures reported previously, the crystal plots were drawn using ORTEP-3 for Windows-version 2020.1. [28], with spheres or 50% probability displacement ellipsoids for all atoms of the compounds being presented.

### 4.3. Docking Simulation for NKA

The NKA crystal structure from 4RET (PDB ID) was used as the receptor, and the receptor structure was prepared by MGLTools to add nonpolar hydrogen and charges [38,45]. Following a previous procedure [20], the structure of each small molecule was built in ChemDraw (ChemDraw 23.1.1 64-bit) and prepared by UCSF Chimera 1.19 [39,46]. The optimization of the 3D structure of the ligand was achieved through UCSF Chimera with the Amber14ffSB force field [47], and charges are computed by ANTECHAMBER [48]. Molecular docking of the isomers and the receptor were implemented on Autodock Vina 1.2.0 [40,41,49] at the docking position of digoxin-4RET. The docking box (26 × 26 × 39 Å^3^) covered the binding pocket formed by α-M1–M6 of NKA, which was focused around the center of D121 and F783 to frame out the region for ligands to explore. Three parallel dockings were operated for each ligand to reduce the randomness of the docking process.

## 5. Conclusions

In the present investigation, docking profiles for conformations with a rotation of the C-3 saccharide moiety or the C-17 lactone of cryptanoside A, digoxin, digoxigenin, and gitoxin and NKA have been investigated. The results showed that conformational variation can lead to docking profiles with NKA being modified, as indicated by the resultant docking scores. In solution, these substituents at the C-3 and C-17 positions of a cardenolide could continue rotating, and thus the resultant rotation conformers in an equilibrium would interact with NKA in a coordinated manner to correlate with a specific bioactivity being observed.

## Figures and Tables

**Table 1:** Docking scores calculated for **1a**–**1f**, **2a**–**2d**, and **3a**–**3e** ^a^.

Conf.	Docking Score		Compd.	Docking Score	
	Average	Minimal		Average	Minimal
**1a**	−11.874	−12.110	**2c**	−11.245	−11.412
**1b**	−13.043	−13.050	**2d**	−11.261	−11.326
**1c**	−12.580	−12.633	**3a**	−8.679	−8.677
**1d**	−12.478	−12.486	**3b**	−8.699	−8.728
**1e**	−10.875	−10.884	**3c**	−9.197	−9.202
**1f**	−10.446	−10.452	**3d**	−11.831	−11.858
**2a**	−10.485	−10.629	**3e**	−12.084	−12.089
**2b**	−10.403	−10.433			

^a^ Docking scores (kcal/mol) calculated from docking **1a**–**1f**, **2a**–**2d**, or **3a**–**3e** to NKA (PDB entry 4RET) by AutoDock Vina.

## Data Availability

The data presented in this study are available on request from the corresponding author, but they are not publicly available due to the requirements of ongoing research.

## Supplementary Materials

The following supporting information can be downloaded at: https://www.mdpi.com/article/10.3390/molecules30244815/s1, Figures S1–S5, rotation conformational effects of selected cytotoxic cardiac glycosides on their interactions with Na^+^/K^+^-ATPase.
