# Supplementary material for: Rotation Conformational Effects of Selected Cytotoxic Cardiac Glycosides on Their Interactions with Na+/K+-ATPase
Source: Molecules. 2025 Dec 18;30(24):4815. doi: 10.3390/molecules30244815 (PMC12736116; doi:10.3390/molecules30244815)
Supplement: Supplementary file 1 [file molecules-30-04815-s001.zip › molecules-4024555-supplementary.pdf]

molecules

Supporting Information

Rotation conformational effects of selected cytotoxic cardiac glycosides on their interactions with Na^+/K^+-ATPase

Yulin Ren [1,‖], Peirun Yang [2,‖], Judith C. Gallucci [1], Can Wang [2], Xiaolin Cheng [1], Sijin Wu [2] and A. Douglas Kinghorn [1,*]

1 Division of Medicinal Chemistry and Pharmacognosy, College of Pharmacy, The Ohio State University, Columbus, OH 43210, United States; ren.41@osu.edu (Y.R.); gallucci.1@osu.edu (J.C.G.); cheng.1302@osu.edu (X.C.)
2 Wisdom Lake Academy of Pharmacy, Xi'an Jiaotong-Liverpool University, Suzhou 215123, Jiangsu Province, People's Republic of China; ucnvpya@ucl.ac.uk (P.Y.); canwang2004@163.com (C.W.); sijin.wu@xjtlu..edu.cn (S.W.)
‖ Equal contributions
* Correspondence: kinghorn.4@osu.edu (A.D.K.)

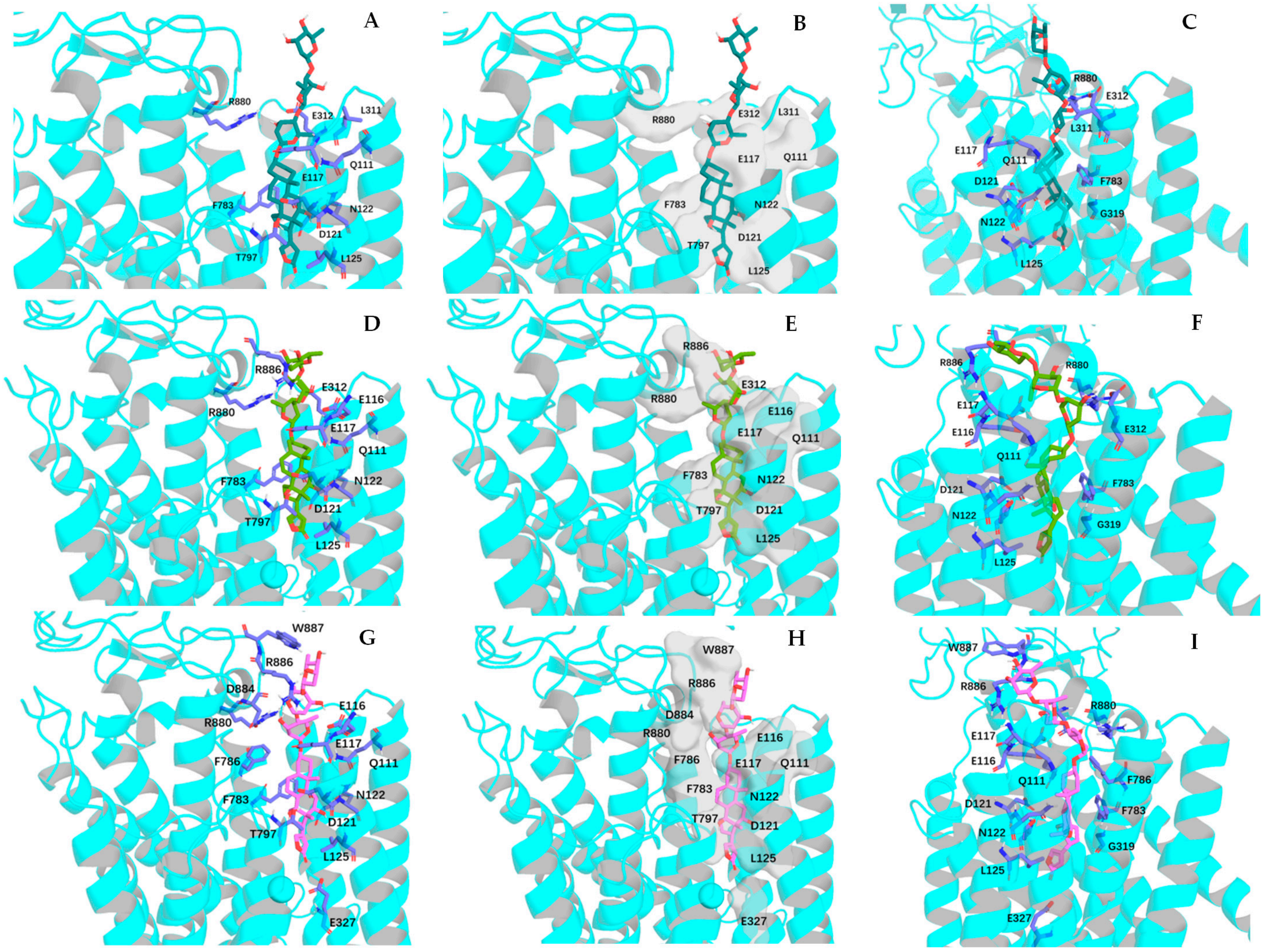

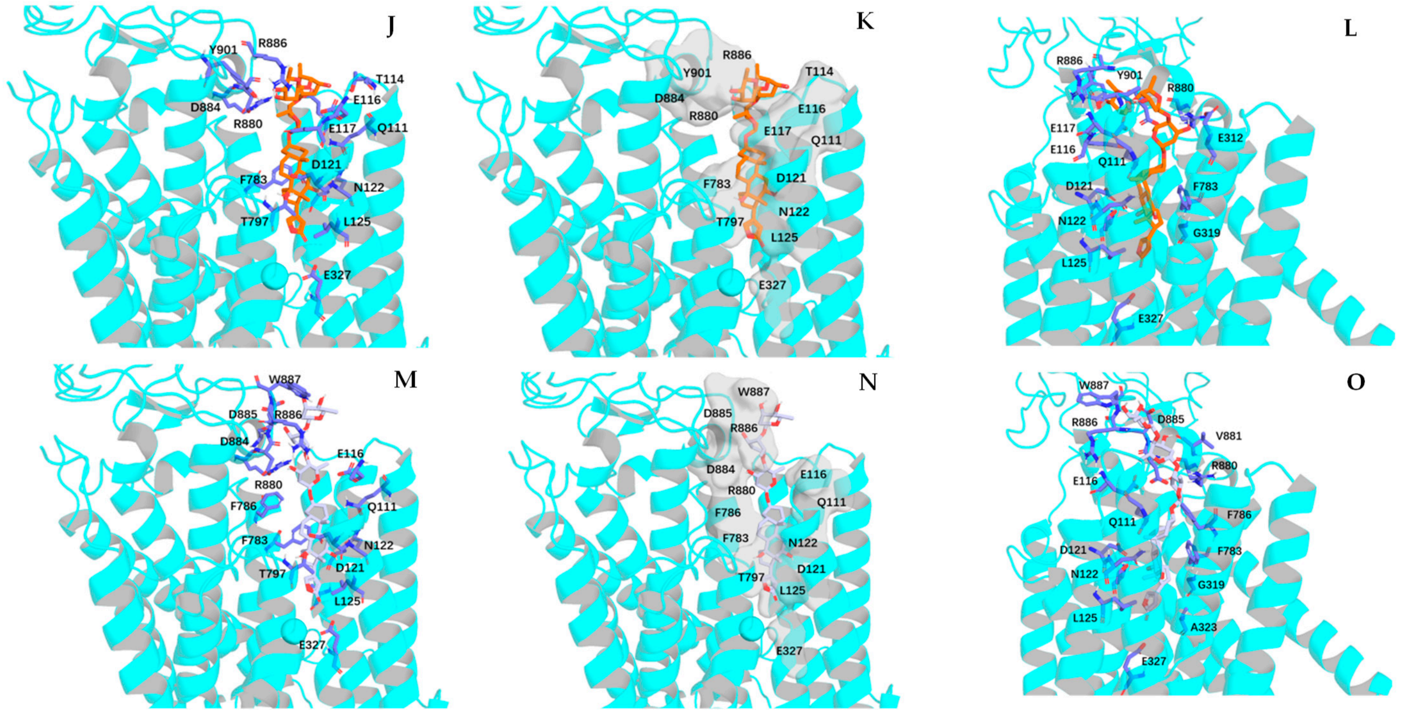

Figure S1. Docking profiles for digoxin-4RENT (deep teal), **1a** (green), **1b** (magenta), **1c** (orange), and **1d** (gray) and NKA. A, D, G, J, and M, the interacting residues are represented by slate sticks; B, E, H, K, and N, the interacting residues are represented by gray surfaces; C, F, I, L, and O, the perspectives were obtained by a 120-degree-rotation of NKA to the left, and the interacting residues are represented by slate sticks.

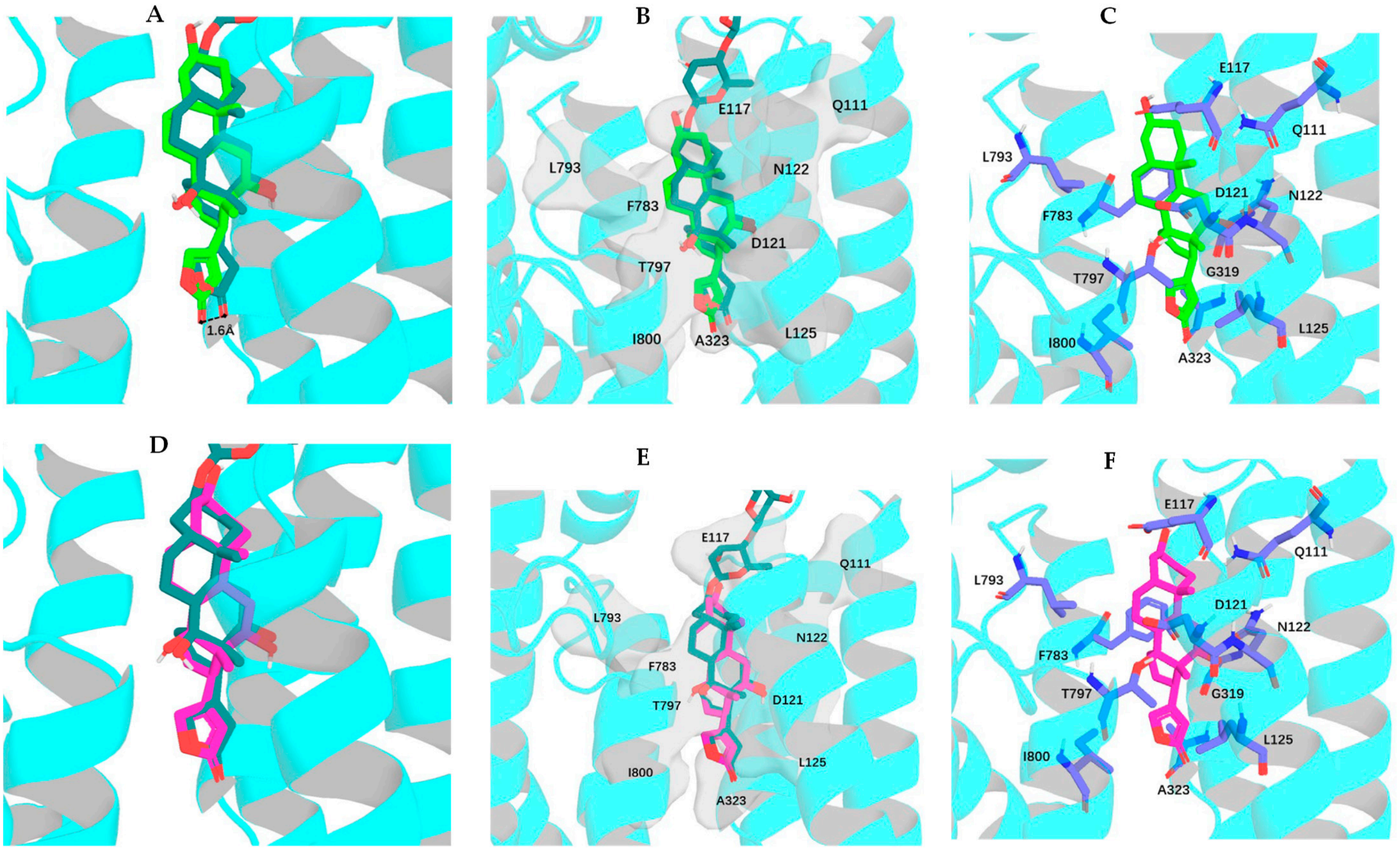

Figure S2. Docking profiles for digoxin-4RENT (deep teal) and **1e** (green) and **1f** (magenta) and NKA. A and D, overlapped docking profiles for digoxin-4RENT, **1e**, or **1f** and NKA, with no interacting residues represented; B and E, overlapped docking profiles for digoxin-4RENT, **1e**, or **1f** and NKA, with the interacting residues represented by gray

surfaces; C and F, docking profiles for **1e** or **1f** and NKA, with the interacting residues represented by slate sticks (digoxin-4RENT, deep teal; **1e**, green; **1f**, magenta.

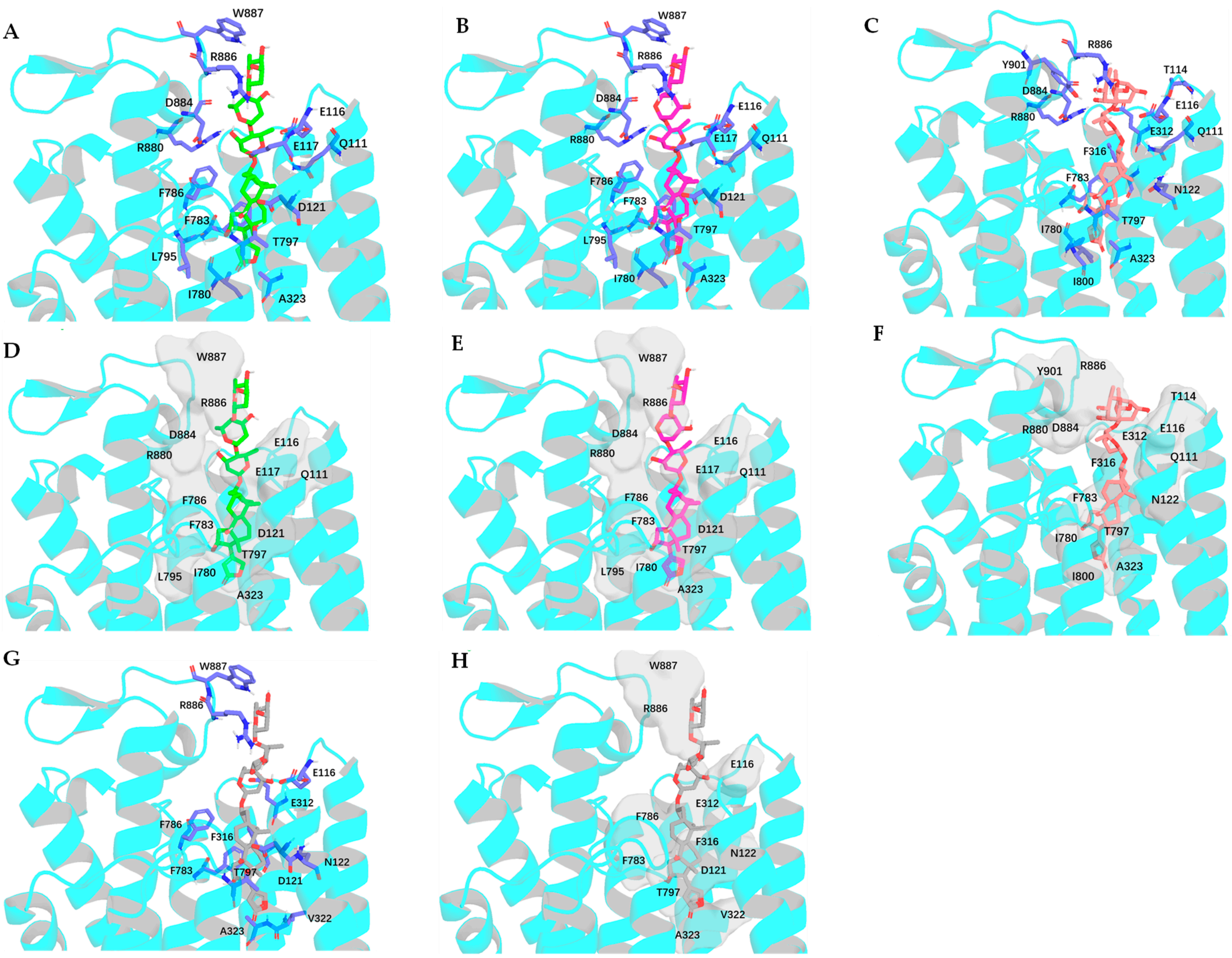

Figure S3. Docking profiles for **2a** (green), **2b** (magenta), **2c** (salmon), and **2d** (gray). A, B, C, and G, the interacting residues are represented by slate sticks; D, E, F, and H, the interacting residues are represented by gray surfaces.

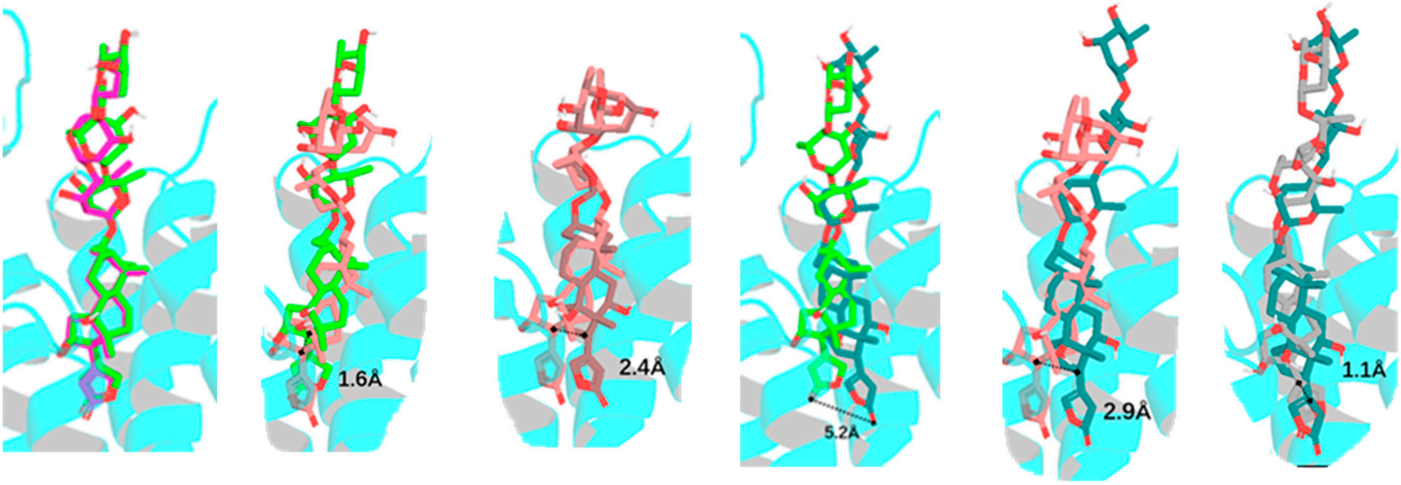

Figure S4. Overlapped docking profiles for digoxin-4RET, **1c**, or **2a–2d** and NKA (**2a,** green; **2b,** magenta; **2c**, salmon; **2d,** gray; **1c**, brown; **and** digoxin-4RET, deep teal).

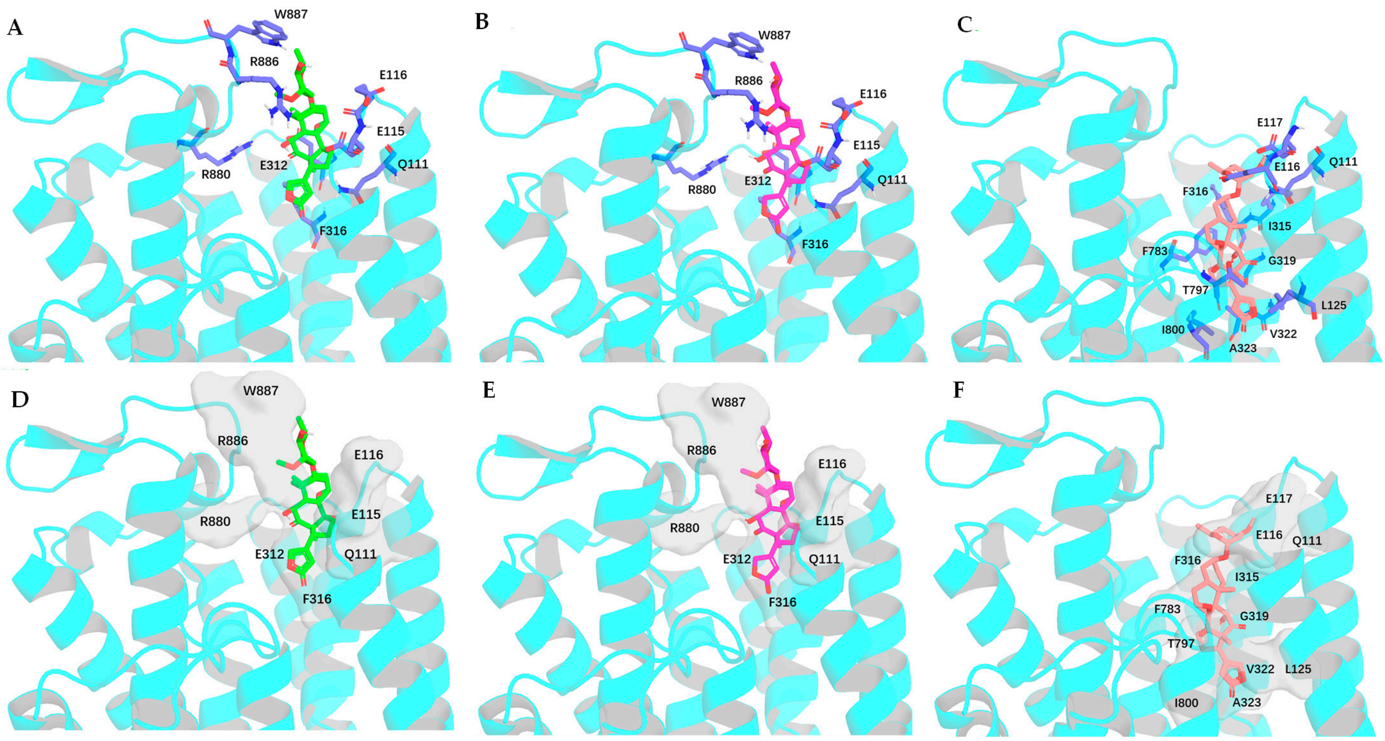

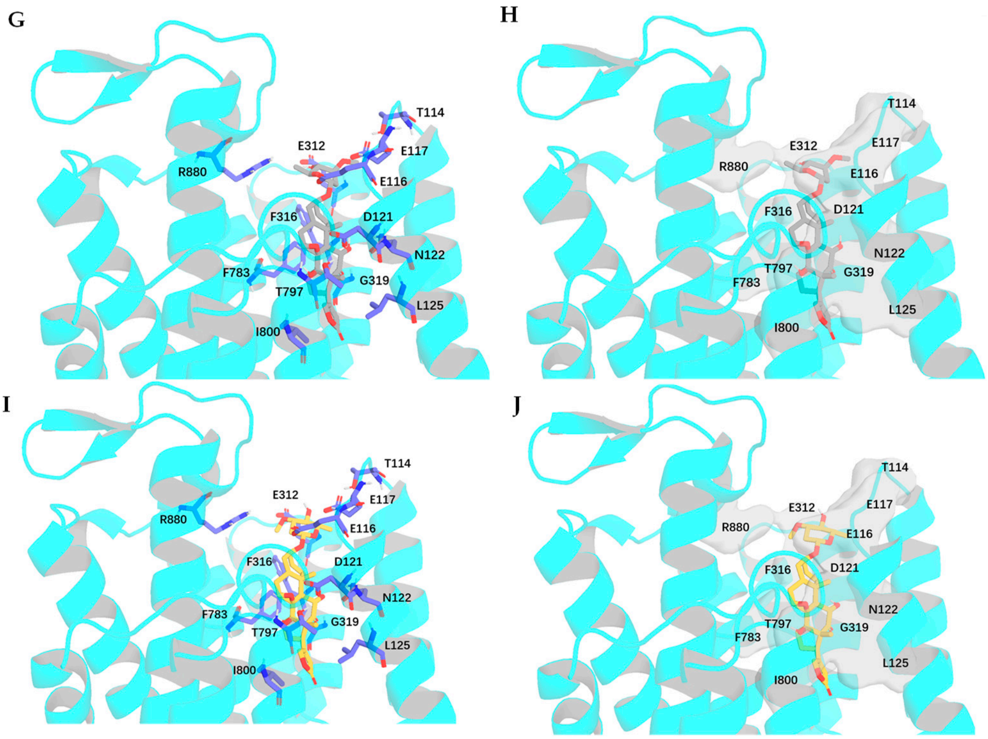

Figure S5. Docking profiles for **3a** (green), **3b** (magenta), **3c** (salmon), **3d** (gray), and **3e** (yellow). A, B, C, G, and I, the interacting residues are represented by slate sticks; D, E, F, H, and J, the interacting residues are represented by gray surfaces.
